# All-Inorganic Intumescent Nanocoating Containing Montmorillonite Nanoplatelets in Ammonium Polyphosphate Matrix Capable of Preventing Cotton Ignition

**DOI:** 10.3390/polym8120430

**Published:** 2016-12-10

**Authors:** Jenny Alongi, Federico Carosio

**Affiliations:** 1 Dipartimento di Chimica, Università degli Studi di Milano, Via Golgi 19, 20133 Milano, Italy; 2 Dipartimento di Scienza Applicata e Tecnologia, Politecnico di Torino, Alessandria Site, Viale Teresa Michel 5, 15121 Alessandria, Italy; federico.carosio@polito.it

**Keywords:** cotton, flame retardancy, combustion, intumescence, sodium cloisite, APP

## Abstract

In the present manuscript a new concept of completely inorganic intumescent flame retardant nanocoating comprised of sodium montmorillonite nanoplatelets embedded in an ammonium polyphosphate matrix has been investigated using cotton as model substrate. The coating, deposited by multistep adsorption from diluted water-based suspensions/solutions, homogenously cover each cotton fibers with average thicknesses below 50 nm and add-on up to 5% in weight. Combustion characterization evidences the interesting properties: indeed, the so-treated fabrics reached self-extinguishing during horizontal flame spread tests. Furthermore, when the coating add-on reaches 5%, no ignition has been observed during cone calorimetry tests under 35 kW/m^2^ heat flux. Residue analyses pointed out the formation of an expanded all-inorganic coating capable of greatly improving char formation by exerting barrier function towards volatile release and heat transfer.

## 1. Introduction

In recent years, the demand for new and sustainable materials has grown and spread in several research fields with the aim of replacing old and inefficient materials concepts with innovative and efficient solutions. In particular, the design of fire safe materials is an area of great concern since the safety and efficiency of conventionally adopted chemistry have been questioned due to perceived human and environment hazards (e.g., halogen-based compounds have been found in the food chain, dangerously ending in the bodies of animals and humans) [1,2,3]. Initial countermeasures to this problem have led to restrain the use of some flame-retardants (FRs) and to start a campaign aiming to evaluate the benefit to danger ratio for the remaining chemicals [4]. In this context, finding non-toxic and high-performing fire retardant solutions is of great industrial and scientific interest.

Nanotechnology represents a possible tool for achieving such goal. Indeed, in the field of materials science, nanomaterials have demonstrated to possess superior properties due to the achieved nanostructures. One clear example of nanostructured materials is represented by polymer-layered nanocomposites; this class of materials has demonstrated remarkable improvements in mechanical strength, oxygen barrier properties and flammability with respect to neat polymers [5]. For instance, the use of lamellar shaped nanoparticles allowed for obtaining peculiar gas barrier and flame retardant properties; indeed, in the former case, when homogeneously dispersed, nanoparticles would create a tortuous path capable of slowing down gas molecule diffusions through polymer matrix while in the latter they would allow for the formation of an inorganic barrier by cumulating on top of a burning polymer and reducing the combustion kinetics [6]. Such results were achieved with low amounts of inorganic filler (usually below 5 wt %) with respect of conventional polymer micro-composites and were tightly bonded for obtaining a nanostructure in which almost each nanoparticle is isolated from the other ones. However, the nanostructuring was not easy to achieve as a lot of efforts during processing and time-consuming modifications of the nanoparticles had to be made in order to homogeneously disperse the nanoparticles within polymer matrix [7].

Recent literature clearly demonstrates that it is possible to overcome such problems, specifically in the field of gas barrier and flame retardancy, by changing the approach towards nanostructuring [8,9]. In this concept, nanoparticles are removed from the bulk in order to be deposited or assemble on the surface, thus resulting in a nanoscale or nanostructured coating capable of greatly improving the performances of the coated polymer. For instance, a PLA (poly(lactic acid))-film coated with efficiently organized nanoplatelets (ideally aligned parallel to the surface and perpendicularly to the gas flux) may reach a oxygen permeability several orders of magnitude lower than that of neat polymer with a substantial improvement of barrier properties with respect to bulk nanocomposites [10,11]. On the other hand, the surface approach for flame retardancy has been demonstrated as a facile and straightforward path to impressive results. This path has been aided by different surface modification techniques based on the deposition from aqueous based suspensions of nanoparticles such as the Layer-by-Layer assembly (LbL) or the simpler nanoparticle adsorption [9,12,13]. In both approaches, the substrate is exposed to a nanoparticle suspension in order to have the adsorption on the surface. This can be performed one time such as in nanoparticle adsorption or multiple times as in LbL assembly. By relying on the electrostatic attractions occurring between nanoparticles and polyelectrolytes in water, the latter approach deposits differently charged species at each adsorption step in order to growth a coating by stacking negatively and positively charged layers [14,15]. Due to the availability of different nanoparticles and polyelectrolytes, many FR actions have been targeted through years. The first and most simple coatings were assembled for obtaining a completely inorganic layer made of nanoparticles; to this aim, sodium montmorillonite or silica nanoparticles have been used on fabrics (cotton and polyester) or thick bulk polymers (polycarbonate (PC), polyamide (PA) and polyester (PET)) [16,17,18,19]. The collected results showed the efficiency of this approach; indeed, the coatings were able to suppress the melt-dripping of PC and PET and to considerably slow down the combustion kinetics of these substrates, often allowing for an unexpected increase in the time to ignition (TTI) [17]. The latter result can be considered unexpected as it is in evidenced contrast with the behavior of nanoparticles observed in bulk nanocomposites where their inclusion in a polymer matrix almost systematically reduced TTI [20].

Through years of research, more complicated coating compositions have been experimented and proven successful [21,22,23]. Of particular interest are the coatings based on the concept of intumescence; their main FR action is due to the formation of an expanded charred structure on the surface of the burning polymer with consequent reduction of heat transmitted from the flame and combustible volatile release [24,25]. This mimics the FR of classical intumescent coatings that are macroscopically bigger reaching several hundreds of microns in thickness [26]. Intumescence in nanostructured coatings can be achieved by ensuring the presence of an acid source, a carbon source and a blowing agent within the coating structure; a simple example is represented by chitosan (CH)/ammonium polyphosphate (APP) coatings where CH acts as carbon source and APP provides phosphoric acid and ammonia as blowing agent [27]. Intumescent coatings normally provided better results than nanoparticle containing ones; as an example, cotton can achieve self-extinguishment behavior during flammability tests only with the deposition of a polyallylamine/polyphosphate or starch/polyphosphate coating [28,29]. The FR action of this kind of coatings has been improved by the inclusion of nanoparticles, thus resulting in a hybrid organic expanded structure reinforced by the inorganic particles. However, if one flaw has to be pointed out, the presence of the organic expanded part represents a weakness of the protective coating as from one hand it can be easily oxidized and from the other hand it possess weak mechanical properties making it prone to collapsing as a consequence of convective motes.

In the present paper we are targeting this flaw by trying to remove the organic part while maintaining the intumescent features of the coating. To this aim, we aim to the deposition of a coating containing sodium montmorillonite (MTM) nanoparticles embedded within an ammonium polyphosphate continuous matrix in order to produce an inorganic expandable structure. The coating concept is derived from the practical observation of what occurs when a mixture of the MTM and APP powders is exposed to a radiant heat flux typical of developing fires. Indeed, the increased temperature triggers the APP dissociation with the release of ammonia and the production of polyphosphoric acid that reacts with MTM nanoplatelets forming of a silicoalluminophosphate expanded structure (see Figure S1 in Supplementary Materials). This and other possible interaction reactions have been already reported in the literature for APP/MTM containing polymers and have been ascribed as one of the reasons for the improved flame retardancy achieved by the combination of the two components with respect to the single constituents [30,31]. Here, we use an easy multi-step adsorption process for the deposition of APP/MTM coatings in order to improve cotton FR properties. Cotton has been selected as model substrate for the evaluation of the coating performances because of its hydrophilic nature and well known degradation process. The morphology of treated and untreated fabrics has been investigated by electron microscopies, the changes in thermal stability have been evaluated by thermogravimetric analyses (in inert and oxidative atmospheres) and the achieved FR characteristics have been assessed by horizontal flame spread tests and cone calorimetry. Finally, the residues collected at the end of the FR tests have been analyzed and a mechanism for the observed FR behavior has been proposed.

## 2. Materials and Methods

### 2.1. Materials

Cotton with an area density of 100 g/m^2^ was purchased from Fratelli Ballesio S.r.l. (Torino, Italy). Prior to deposition, cotton fabrics were washed with water and Marseille soap, ethanol and then diethyl ether. After the washing steps, fabrics were dried in an oven at 70 °C for 1 h.

The sodium montmorillonite was purchased from Southern Clays Products Inc. (Gonzales, TX, USA) and employed in 1 wt % water suspension. The suspension was kept under magnetic stirring for 24 h and then centrifuged at 4400 rpm for 5 min in order to remove aggregates, resulting in a final concentration of 0.7 wt %. 

Ammonium polyphosphate (PHOS-CHEK^®^ P30, purchased from ICL Performance Products Inc., Milano, Italy) was used for preparing 1 wt % water solution that was kept under magnetic stirring for 24 h prior to use. The 18.2 MΩ deionized water supplied by a Q20 Millipore system (Milano, Italy) was employed for both APP solutions and MTM suspensions.

### 2.2. Coating Deposition

Cotton fabrics were alternately dipped in the APP solution and then in MTM suspension. In between each adsorption step, the fabrics were squeezed using a Padder model FL300 produced by Gavazzi S.r.l (Bergamo, Italy) and dried in a convection oven at 80 °C for 30 min, mimicking an impregnation pad-dry industrial treatment. The dipping time was set at 5 min for the first couple of adsorption steps; subsequent steps were achieved after 1 min. The alternate dipping/padding/drying cycle was repeated 2 or 4 times in order to achieve final coating add-ons of 2.5% and 5%, respectively. In the following, coated samples are coded as % add-on APP/MTM.

### 2.3. Characterization

Scanning Electron Microscopy: the change in surface morphology of treated cotton with respect to untreated one was evaluated using a Field-Emission Scanning Electron Microscopy (FE-SEM) on a ZEISS, FEG model MERLIN. A LEO-1450VP Scanning Electron Microscope (Carl Zeiss Microscopy GmbH, Jena, Germany) equipped with an X-ray probe (INCA Energy Oxford, Oxfordshire, UK, Cu-Kα X-ray source, *k* = 1.540562 Å) was used to perform elemental analysis (Energy Dispersive Spectroscopy, EDS). In both cases untreated and treated cotton fabrics were cut (10 × 10 mm^2^), fixed to conductive adhesive tapes and either chromium- (FE-SEM) or gold- (SEM) metallized prior to imaging.

X-ray diffraction: X-ray diffraction spectra (XRD) were collected on 30 × 30 × 0.5 mm^3^ samples with a Philips X’Pert-MPD diffractometer (PANalytical, Eindhoven, The Netherlands, Cu-Ka radiation, *k* = 1.540562 Å; step size: 0.02; step time: 2 s).

Thermal stability: A TAQ500 thermogravimetric balance (TA-Instruments, Milano, Italy) was used for thermogravimetric analyses. The tests were performed from 50 to 800 °C (heating rate of 10 °C/min) in both nitrogen and air (60 mL/min) on 10 mg samples placed in open alumina pans. The following parameters were assessed: *T*_onset 5%_ (temperature at which a 5 wt % weight loss is registered), *T*_max_ (temperature at which the maximum weight loss is registered), residue at *T*_max_ and 800 °C. The experimental error was 0.5% on the weight and 1 °C on the temperature. Thermogravimetric (TG) and derivative (dTG) curves were reported.

Horizontal flame spread tests: The reaction to the flame application of the prepared samples was evaluated in horizontal configuration. During the test the sample (100 × 50 mm^2^) was placed in a metallic frame and tilted 45° with respect to its longer axis, then a 20 mm blue methane flame is applied to its short side for 3 s in order to ignite it. Parameters such as burning time, afterglow times and final residue were registered during the test. At least three tests were performed for each formulation.

Cone calorimetry: An oxygen consumption cone calorimeter (Fire Testing Technology, FTT) was employed to investigate the combustion behavior of square samples (100 × 100 mm^2^) under 35 kW/m^2^ in horizontal configuration. Tests were performed following the ISO 5660 standard implementing the optimized procedure for textiles described elsewhere [32]. The following parameters were registered: Time To Ignition (TTI, (s)), peak of Heat Release Rate (pkHRR, (kW/m^2^)), Total Heat Release (THR, (MJ/m^2^)) and final residue. At least three tests were performed and standard deviation (σ) was calculated as experimental error; for non-igniting samples the test was repeated five times.

Fourier Transformed Infrared Spectroscopy in Attenuated Total Reflectance (FT-IR ATR): Spectra were collected at room temperature (range 4000–700 cm^−1^, 16 scans and 4 cm^−1^ resolution) using a Frontier FT-IR/FIR spectroscopy (Perkin Elmer, Milano, Italy) in ATR configuration, equipped with a diamond crystal (depth of penetration 1.66 µm, as stated by the producer).

Raman spectroscopy: Analyses were performed on an InVia Raman Microscope (argon laser source 514 nm/50 mW, Renishaw S.p.A., Torino, Italy) coupled with a Leica DM 2500 optical microscope (Leica Microsystems S.r.l., Milano, Italy).

## 3. Results

### 3.1. Coating Morphology on Cotton

**Figure 1:**
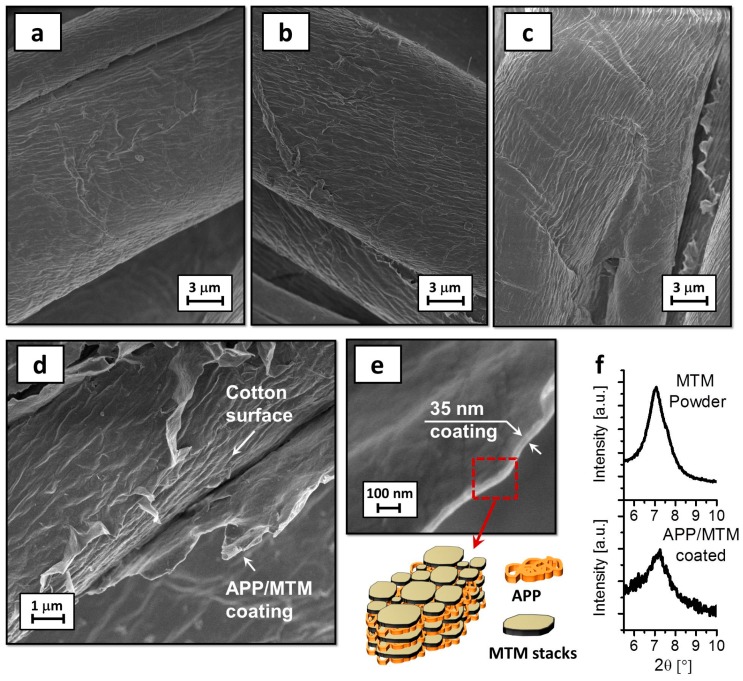
FE-SEM (Field-Emission Scanning Electron Microscopy) micrographs of: neat cotton (**a**); 2.5% APP/MTM sample (**b**); 5% APP/MTM sample (**c**); details of 5% APP/MTM (**d**,**e**); and XRD spectra of neat MTM and 5% APP/MTM fabric (**f**).

Coating morphology on cotton fibers has been carefully investigated by FE-SEM observation combined with XRD diffraction. Figure 1 reports micrographs of uncoated and APP/MTM-coated cotton and XRD spectra performed on neat MTM powder and 5% APP/MTM sample.

Neat cotton shows the typical morphology of a natural fiber with a rough and irregular surface. When cotton is coated by APP/MTM, no immediate change in morphology can be detected. Indeed, by a direct comparison of the uncoated and coated fibers, it is really difficult to establish whether a coating has been deposited.

The presence of the coating has been revealed by investigating sites where, as a consequence of deformations, the coating is partially detached from cotton surface.

As reported in Figure 1, high magnification micrographs point out the presence of a very thin coating that averages 35 nm for 5% APP/MTM samples. Similar investigations are possible on 2.5% APP/MTM and result in thinner and less homogenous coatings, which thickness is more difficult to evaluate (see Figure S2 in Supplementary Materials).

The weight gain after the deposition was found to be around 2.5 and 5 wt %; furthermore, the deposited nanocoating had no impact on fabric hand and color. From the micrographs collected in Figure 1, different information can be gathered: the coating is really thin and when deposited on cotton fibers is capable of literally reproducing the surface irregularities of the original fibers and MTM nanoplatelets are adsorbed on the surface with a strong in-plane orientation.

The last statement is also confirmed by XRD measurements. As reported in the interpherogram, neat MTM shows the characteristic peak at 7.0° related of the basal spacing between each MTM nanoplatelet, which is consistent with the literature [33,34]. APP/MTM coated fabrics displayed a diffraction peak in the same region indicating that MTM is adsorbed as stacks consisting of several nanoplatelets laying parallel on the surface and are held together by APP in a “brick and mortar-like structure”, as schematized in Figure 1 [35].

EDS analyses performed on so-coated samples revealed the presence of elements characteristic of APP (phosphorous) and MTM (silicon), further confirming the presence of both reagents within the coating (see Figure S3 in Supplementary Materials). By evaluating the different Si and P percentages, it is possible to semi-quantitatively estimate the coating composition for each sample; 2.5% APP/MTM contains 70% APP and 30% MTM while this proportion is basically inverted for 5% APP/MTM which contains 30% APP and 70% MTM. Such difference might be related to the interactions between the negative phosphate groups of previously adsorbed APP and the positive edges of adsorbing MTM with the subsequent release of ammonium ions eventually employed for the production of stacked MTM layers. Such interaction would be possible only after the deposition of few (2–3) APP layer and thus be more apparent after eight deposition steps rather than four. However, while this explanation seems in accordance with XRD analyses (Figure 1f), in the absence of more detailed characterization the proposed adsorption mechanism remains a speculation. Undoubtedly, composition difference, along with the total coating add-on, could play an important role during cotton thermal degradation and flame retardancy.

### 3.2. Thermal and Thermo-Oxidative Stability

**Figure 2:**
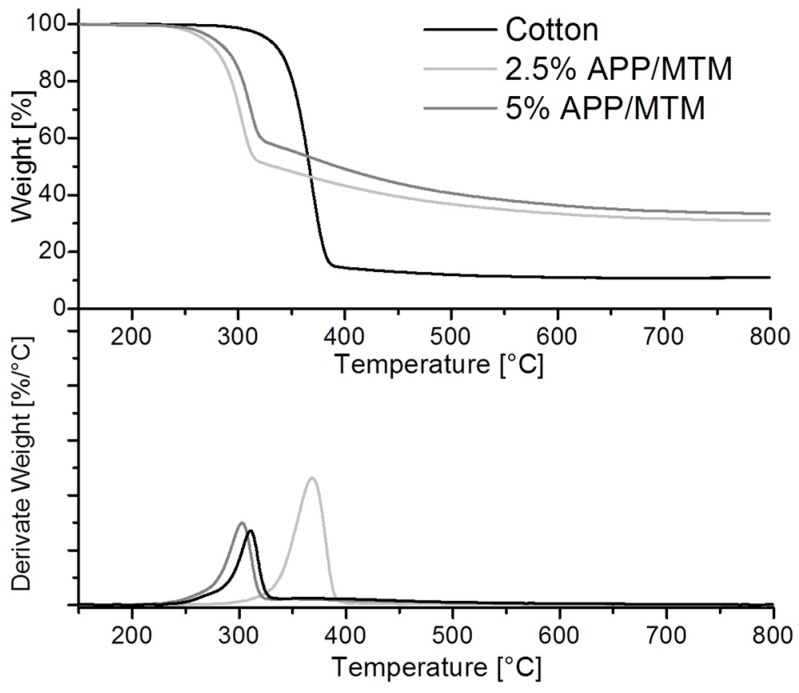
Thermogravimetric (TG) and derivative (dTG) curves of untreated and treated cotton fabrics in nitrogen.

The thermal and thermo-oxidative stability of untreated and coated samples was assessed by thermogravimetric analyses in nitrogen and air, respectively. The aim is to obtain useful information concerning the effects of the deposited coating on the degradation pathways of cotton fabrics. First, only thermal degradation is evaluated in nitrogen environment. Figure 2 reports TG and dTG curves of untreated and treated samples and Table 1 collects temperature and weight data obtained from these analyses.

The weight loss of cotton as a function of the temperature can be mostly related to the thermal degradation of cellulose, which is well known as well as its mechanism is already established. In nitrogen, the thermal decomposition of cellulose normally occurs by one step in between 300 and 400 °C and is the result of two competitive pathways, one involving the depolymerization of glycosyl units to volatile products (mainly levoglucosan, furan and furan derivatives) and the other involving the decomposition of the same units into thermally stable aromatic char (final residue evaluated at 800 °C, 11%) [36].

Treated fabrics still show a one-step thermal degradation. However, as clearly observable from Figure 2 the presence of the coating induces a strong anticipation in the degradation process, (see *T*_onset5%_ and *T*_max_ values in Table 1). This anticipation, more precisely defined “sensitization”, is well known and associated to APP decomposition that producing phosphoric acid at high temperature favors the cellulose decomposition towards char formation [37,38].

Interestingly there is an inverse proportionality between the coating add-on and the anticipation observed in TG curves; this can be explained by taking into account the different coating compositions, as previously observed from elemental analysis. Indeed, the P/Si ratio for 2.5% APP/MTM treated samples is higher than for 5% sample, thus indicating a higher amount of APP within the coating with a consequent increase of the anticipating effect.

On the other hand, the effect of MTM, the presence of which is proportionally higher in 5% APP/MTM coatings, also has to be considered. Due to the lamellar chemical nature of these nanoparticles and the preferential orientation achieved through the deposition as observed in Figure 1, MTM can easily exert a barrier function towards the release of volatile products, thus balancing the anticipation ascribed to APP. This hypothesis is in agreement with the literature [35]. The latter combination of the two counterparts is beneficial and indeed provides the highest residue at *T*_max_ and at 800 °C, nearly tripling the amount left by neat cotton (compare residues at 800 °C in Table 1).

**Figure 3:**
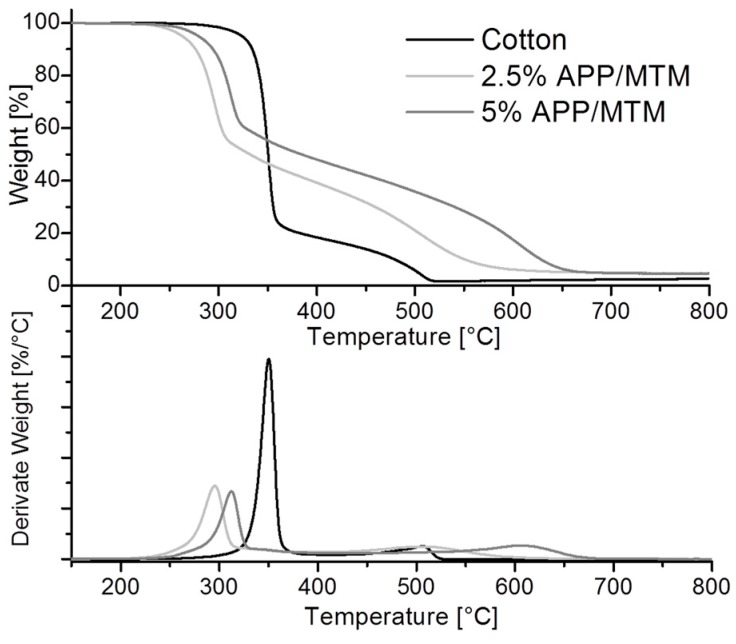
Thermogravimetric (TG) and derivative (dTG) curves of untreated and treated cotton fabrics in air.

The thermo-oxidative stability of untreated and treated cotton fabrics has been assessed in air, thus evaluating the effects of an increasing temperature in an oxidizing environment. Figure 3 reports TG and dTG curves in air of untreated and treated samples and Table 2 collects temperature and weight data calculated from the plots.

The thermal oxidation of cotton normally takes place in two definite steps: the first one between 300 and 400 °C is related to the formation of both volatiles and al aliphatic char (18%), while the second is due to the almost complete oxidation of the char with the release of CO and CO_2_ [39,40,41]. Similar to what observed in nitrogen, treated fabrics showed anticipation inversely proportional to the coating add-on (compare *T*_onset5%_ values in Table 2).

Of particular interest is the coating shielding effect towards oxygen during the second degradation step; here, the 5% APP/MTM sample maintains 40% residue up to 550 °C and achieves the highest delay in the second degradation step, as confirmed from T_max2_ value that is shifted to 600 °C with an increase of 100 °C with respect to unmodified cotton (compare *T*_max2_ values in Table 2). This effect can be ascribed to the formation of a protective coating capable of greatly postponing and slowing down the oxidation of the residue produced during the first degradation step.

### 3.3. Horizontal Flame Spread Tests

**Figure 4:**
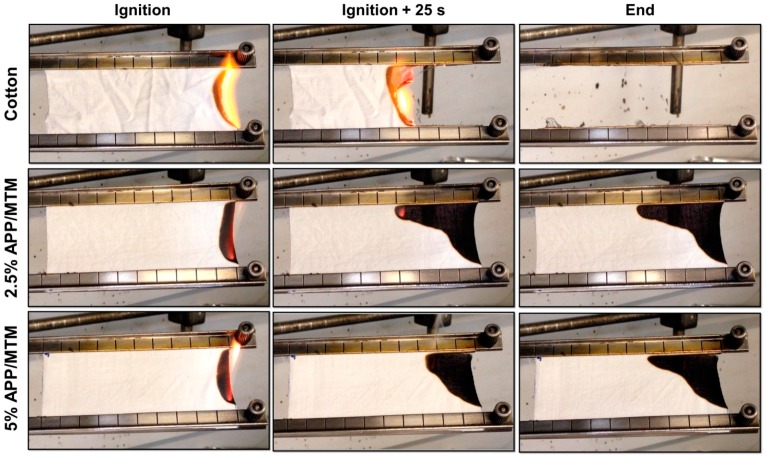
Snapshot of flame-spread tests of untreated and treated cotton fabrics.

Flammability test in horizontal configuration has been employed to investigate the reaction of untreated and treated fabrics to a direct flame application. This test assesses the propensity of a material to initiate a fire and represents a fundamental test in the field of flame retardancy. Figure 4 reports snapshots of uncoated and coated fabrics during the test and Table 3 reports the collected parameters.

Upon flame application, untreated cotton immediately ignites and burns with flames that, fed by the combustible degradation products of cotton, spread towards the opposite site of sample at an almost constant speed. As the flame reaches the end of the sample, it vanishes and the remaining charred cotton fibers are further consumed by a solid-state oxidation characterized by red incandescence known as afterglow. The high temperatures reached during the afterglow are still capable of spreading the fire to other ignitable materials, thus posing an additional, although smaller than flames, risk to safety.

The coating can significantly modify the burning behavior of cotton. Indeed, both treatments were able to stop the propagation of the flame achieving a self-extinguishing behavior, thus resulting in very high residues. In detail, upon flame propagation the coating action as protective barrier and char formation enhancer reduces the release of volatile combustible products; by this way, the combustion cannot be sustained anymore and the flame gradually reduces in size being confined to a smaller and smaller region where, eventually, it vanishes (see Figure 4). Furthermore, any subsequent flame application cannot ignite the sample again.

### 3.4. Cone Calorimetry

Cone calorimetry has been employed in order to evaluate the reaction of uncoated and coated samples to the exposure to a heat flux. The latter is controlled in order to have heat flux values typical of early stage developing fires (i.e., 35 kW/m^2^) [42]. When exposed to the heat flux, samples start to degrade releasing combustible gases that are ignited by a spark positioned above the sample. The time required for reaching ignition is normally referred as time to ignition (TTI). Then, flaming combustion starts and the heat released is calculated by evaluating the oxygen consumed during the process.

**Figure 5:**
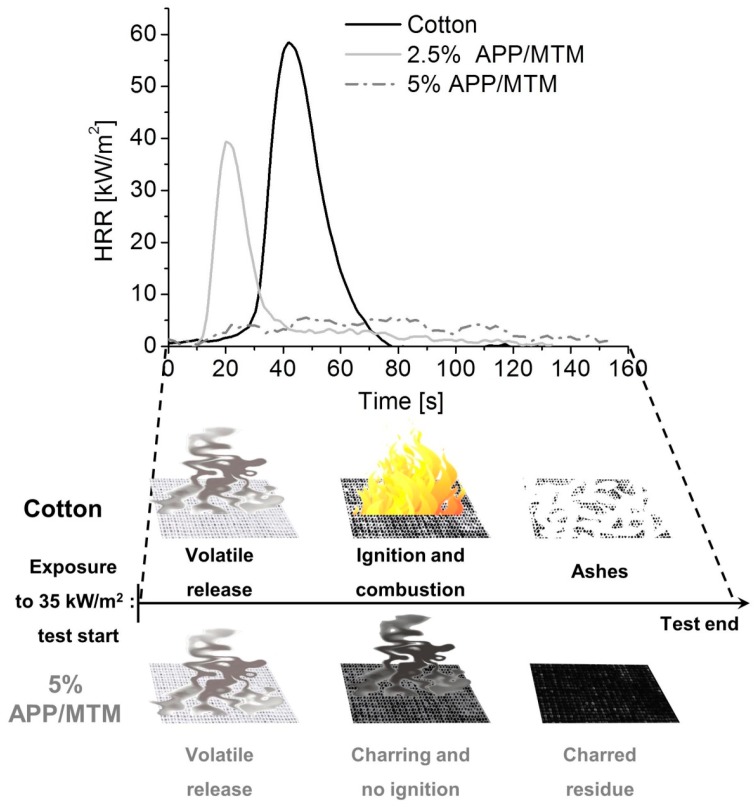
Average Heat release rate (HRR) plots of untreated and treated cotton during cone calorimetry tests and schematic representation of sample behavior under testing.

Heat Release Rate (HRR) plots of untreated and treated samples are reported in Figure 5 together with a schematization of sample behavior during the test. Table 4 reports the collected numerical data.

Unmodified cotton rapidly ignites after 36 s with a quick combustion and an average pkHRR of 61 kW/m^2^, without leaving any residue at the end. The 2.5% APP/MTM shows an anticipation in ignition as TTI is reduced to 22 s; this can be ascribed to the presence of APP that, similar to what observed in TGA, releases phosphoric acid that favors cellulose dehydration, thus resulting in an early production of volatiles with subsequent early ignition. This phenomenon is not considered detrimental, as by this way the production of charred residue is favored despite volatile release [29]. Thus, the total heat release and combustion kinetics are significantly reduced, as demonstrated by HRR plots and THR and pkHRR values reported in Table 4.

Surprisingly, 5% APP/MTM samples showed no ignition at all during this test. Upon exposure to the heat flux, samples start releasing volatile products while simultaneously producing char, as clearly observable by a change in color (from white to black). The latter process, improved by the presence of APP, is combined with the barrier effect exerted by MTM that slows down the gas release. Therefore, by the combination of these two flame retardant actions, the released combustible volatile products are not able to reach the concentration needed for ignition, as schematically depicted in Figure 5. This is also confirmed by TSR (Total Smoke Release) values that show an increase for non-igniting samples (Table 4).

All treated fabrics yielded a compact and coherent residue at the end of the test (see Figure S4 in Supplementary Materials) that, at the higher coating add-on, maintained the original shape of the fabric. Interestingly, it was possible to handle the residues without damaging them (e.g., they could be bent by 180° without breaking), thus indicating that they partially maintained the original mechanical properties.

### 3.5. Residue Analysis and Coating Mechanism

**Figure 6:**
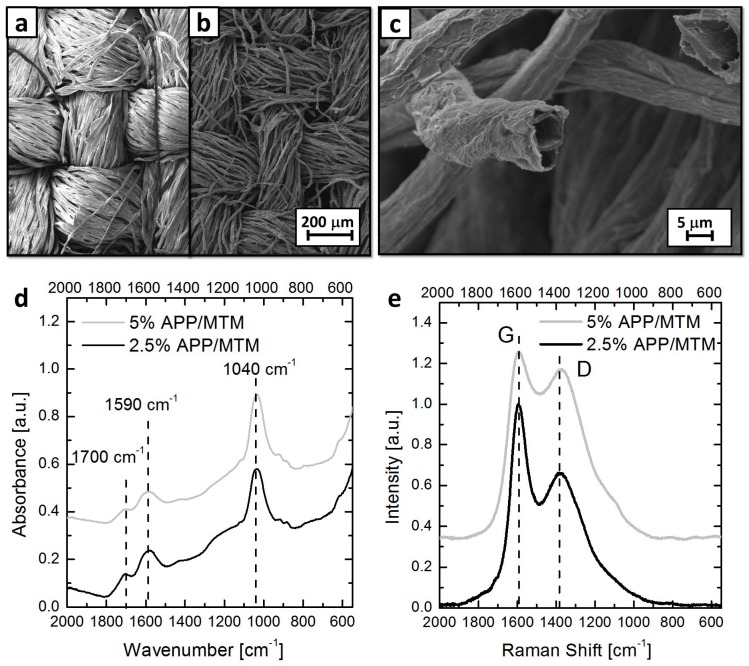
Analyses on residues after cone calorimetry: low magnification SEM (Scanning Electron Microscopy) micrograph performed on unburned (**a**) and burned (**b**) areas of 5% APP/MTM; detail of 5% APP/MTM fibers after combustion (**c**); and FT-IR/ATR and Raman spectra (**d**,**e**). As observable from SEM micrograph, the residues were able to maintain the original texture of the fabric while the single fibers appear damaged and shrunk with respect to the unburned sample (compare Figure 6a,b). In addition, from a closer observation, the single fibers appear to be surrounded by a sort of expanded structure that completely covers them. This structure is resulting from the exposure of the original APP/MTM coating to a heat flux; EDS analysis confirms the composition by pointing out the presence of both P and Si elements with C being the main component (see Figure S5 in Supplementary Materials).

The residues collected at the end of cone calorimetry tests have been analyzed using SEM, FT-IR/ATR and Raman spectroscopies in order to obtain information useful for interpreting the results obtained during flammability and cone calorimetry tests. Figure 6 reports SEM observations performed on 5% APP/MTM, and IR and Raman spectra of both 2.5% APP/MTM and 5% APP/MTM residues.

IR and Raman spectroscopies provide additional information concerning the structure of the carbonaceous structure. Indeed, IR signals related to aromatic char can be found at 1590 cm^−1^ while the signal at 1040 cm^−1^ can be associated to Si–O–Si bonds in MTM nanoplatelets [43]. Furthermore, the presence of a weak peak at 1700 cm^−1^ associated to C=O bonds indicates the partial oxidation of the residues.

Raman spectroscopy gives complementary details concerning the nature of the produced char. As reported in Figure 6e, both residues show two characteristic peaks (namely, G and D bands at 1590 and 1350 cm^−1^, respectively) normally associated to polyaromatic hydrocarbons, further confirming the aromatic nature of the structures produced during combustion [44,45].

**Figure 7:**
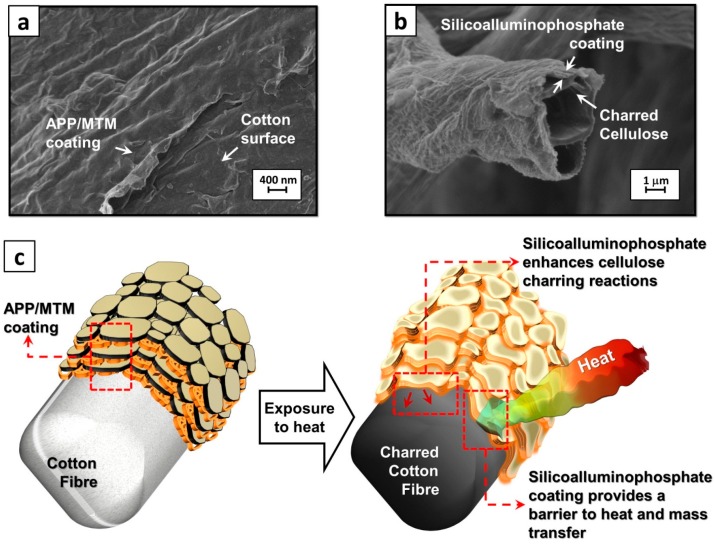
Coating reaction to heat or flame application: FE-SEM micrograph of coating (5% APP/MTM)) before combustion (**a**); SEM micrograph of expanded coating (5% APP/MTM) after combustion (**b**); and schematization of the coating flame retardant action (**c**).

Basing on the achieved results and the mentioned above characterization, it is possible to devise the coating mechanism. To this aim, the coating reaction to heat or flame application is reported in Figure 7 where a FE-SEM micrograph of coating (5% APP/MTM)) before combustion (Figure 7a), SEM micrograph of expanded coating (namely, 5% APP/MTM) after combustion (Figure 7b) and a schematization of the coating flame retardant action (Figure 7c) are depicted.

Upon exposure to a heat flux or flame, APP starts to degrade releasing ammonia and producing phosphoric and polypohosphoric acid [38]. Ammonia swells the MTM stacks deposited within the coating while polypohosphoric acid simultaneously promotes the char formation of cotton, as well known from the literature [37]. In addition, besides reacting with cotton for enhanced char production, APP can also react with MTM nanoplatelets; indeed, clay can favor the dissociation of APP and the phosphoric acid generated can react with MTM nanoplatelets joining them together with the production of a silicoalluminophosphate [30,31]. In the case of the coating, the inorganic expanded structure can also act as physical barrier slowing down mass, oxygen and heat transfer between the flame and the substrate.

## 4. Conclusions

In the present manuscript, a new concept of completely inorganic intumescent flame retardant coating deposited by a multistep adsorption process from diluted water-based ammonium polyphosphate solutions and sodium montmorillonite suspensions has been explored, using cotton as model substrate. The coating covers the surface of each cotton fiber resulting in very thin coatings with thickness below 50 nm where montmorillonite stacks are embedded in a continuous matrix of ammonium polyphosphate. This coating efficiently enhanced the char production from cotton fibers, as observed by thermogravimetric analyses in both nitrogen and air, with impressive results achieved during combustion tests. The coating resulting in a total add-on of 5% with respect to the original fabric mass achieved self-extinguishing during horizontal flame spread tests and prevented ignition of the fabrics when exposed to 35 kW/m^2^ during cone calorimetry test. The flame retardant action has been ascribed to the formation of a silicoalluminophosphate expanded structure capable of on the one hand enhancing the cellulose char-forming ability and on the other hand providing a physical barrier to mass, oxygen and heat transfer between the flame and the substrate. These results demonstrate the potentialities of the concept proposed and developed within this paper and provide a starting point for the further improvements of the coating performances. For instance, the coating stability and durability need to be improved by employing different cross-linking strategies if an application as protective treatment for fabrics has to be foreseen.

## Figures and Tables

**Table 1:** Thermogravimetric data of untreated and treated samples in nitrogen.

Sample	*T* *_onset5%_ (°C)	*T* *_max_ (°C)	Residue at 800 °C (%)
Cotton	327	369	11
2.5% APP/MTM	266	301	31
5% APP/MTM	276	310	34

* From derivative TG curves.

**Table 2:** Thermogravimetric data of untreated and treated samples in air.

Sample	*T* *_onset5%_ (°C)	*T* *_max1_ (°C)	*T* *_max2_ (°C)	Residue at 400 °C (%)	Residue at 800 °C (%)
Cotton	324	350	506	18	2
2.5% APP/MTM	263	296	508	39	4
5% APP/MTM	280	313	607	48	5

* From derivative TG curves.

**Table 3:** Flammability data from horizontal flame tests of untreated and treated cotton fabrics.

Sample	Combustion Rate ± σ (mm/s)	Afterglow	Residue ± σ (%)
Cotton	1.7 ± 0.04	Yes	0
2.5% APP/MTM	1.6 ± 0.30	No	78 ± 5
5% APP/MTM	1.3 ± 0.08	No	85 ± 2

**Table 4:** Cone calorimetry data of untreated and treated samples.

Sample	TTI ± σ (s)	pkHRR ± σ (kW/m^2^)	THR ± σ (MJ/m^2^)	TSR ± σ (m^2^/m^2^)	Residue (%)
Cotton	36 ± 2	61 ± 4	1.0 ± 0.1	25 ± 8	0
2.5% APP/MTM	22 ± 5	38 ± 7	0.38 ± 0.05	13 ± 4	13
5% APP/MTM	N.A. *	N.A. *	N.A. *	40 ± 6	19

* Parameters related to combustion are not available, as samples did not ignite during test.

## Supplementary Materials

The following are available online at http://www.mdpi.com/2073-4360/8/12/430/s1, Figure S1: Images of MTM (a), APP (b) and mixture of APP/MTM (c) after irradiation under 35 kW/m^2^ heat flux in a cone calorimeter. Figure S2: FE-SEM micrographs of defects in 2.5% APP/MTM samples. Figure S3: EDS analyses performed on 2.5% APP/MTM and 5% APP/MTM samples. Figure S4: Images of residues collected at the end of cone calorimetry tests: (a) untreated cotton, (b) 2.5% APP/MTM and (c) 5% APP/MTM samples. Figure S5: EDS analyses performed on 5% APP/MTM residues collected at the end of cone calorimetry tests.
