# Supplementary material for: All-Inorganic Intumescent Nanocoating Containing Montmorillonite Nanoplatelets in Ammonium Polyphosphate Matrix Capable of Preventing Cotton Ignition
Source: Polymers (Basel). 2016 Dec 10;8(12):430. doi: 10.3390/polym8120430 (PMC6432209; doi:10.3390/polym8120430)
Supplement: Supplementary file 1 [file polymers-08-00430-s001.pdf]

Supplementary Materials: All-Inorganic Intumescent Nanocoating Containing Montmorillonite Nanoplatelets in Ammonium Polyphosphate Matrix Capable of Preventing Cotton Ignition

Jenny Alongi and Federico Carosio

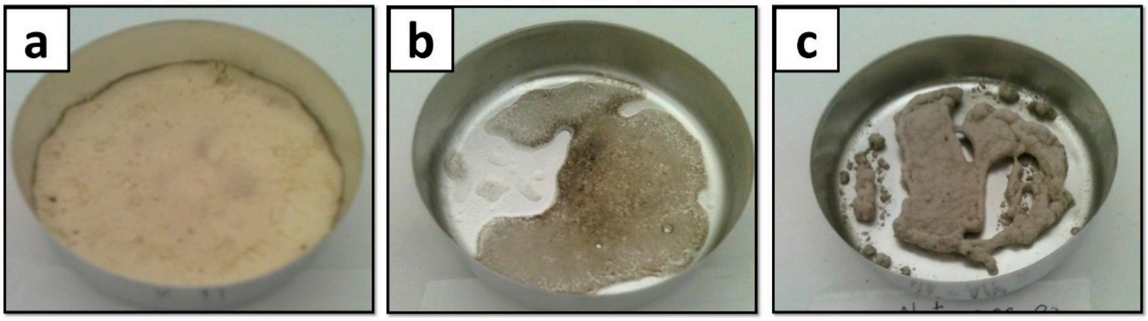

Figure S1. Images of MTM (**a**); APP (**b**) and mixture of APP/MTM (**c**) after irradiation under 35 kW/m² heat flux in a cone calorimeter.

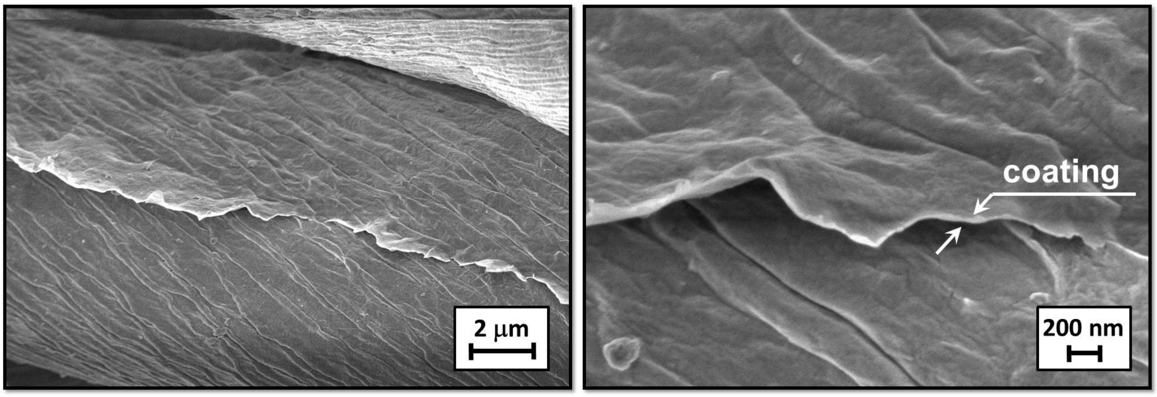

Figure S2. FE-SEM micrographs of defects in 2.5% APP/MTM sample.

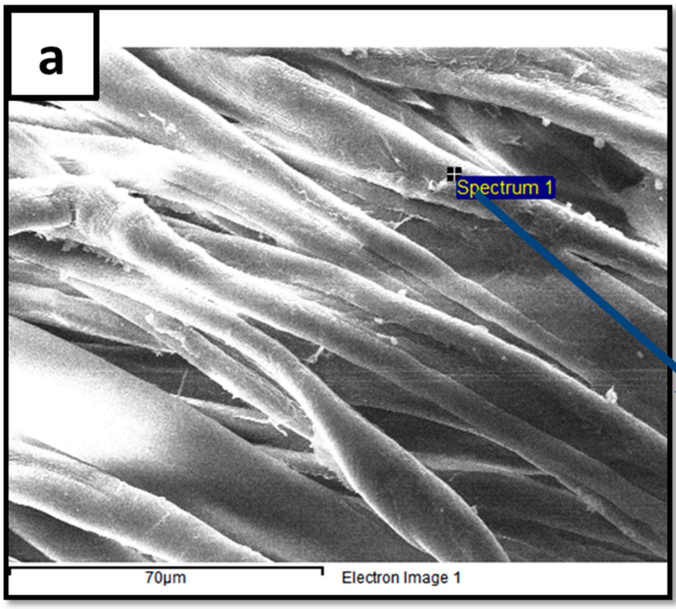

Element	Weight%
C	49.13
O	48.08
Al	0.40
Si	0.49
P	1.90
Total	100.00

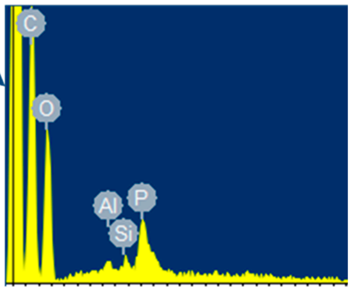

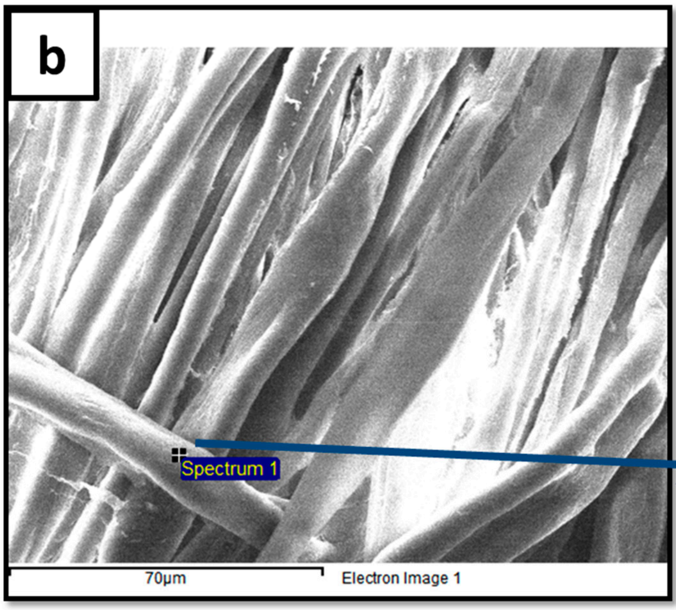

Element	Weight%
C	44.19
O	51.77
Al	0.72
Si	1.99
P	1.33
Totals	100.00

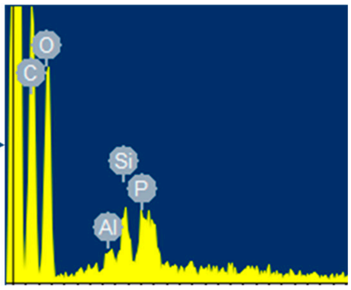

Figure S3. EDS analyses performed on 2.5% APP/MTM (a) and 5% APP/MTM (b) samples.

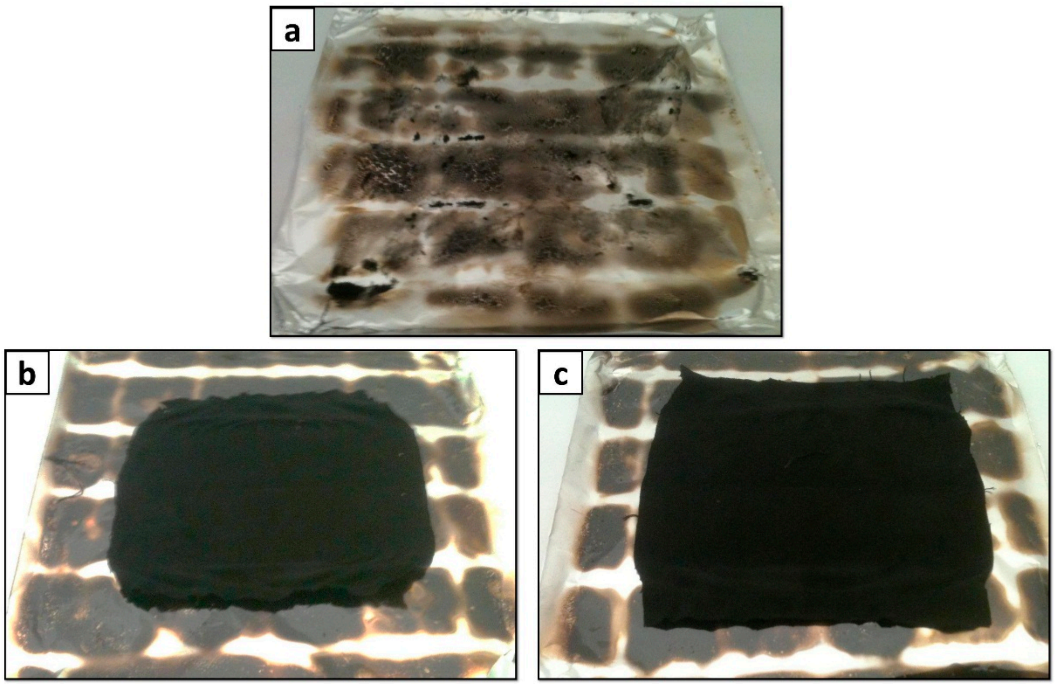

Figure S4. Images of residues collected at the end of cone calorimetry tests: (**a**) untreated cotton, (**b**) 2.5% APP/MTM and (**c**) 5% APP/MTM samples.

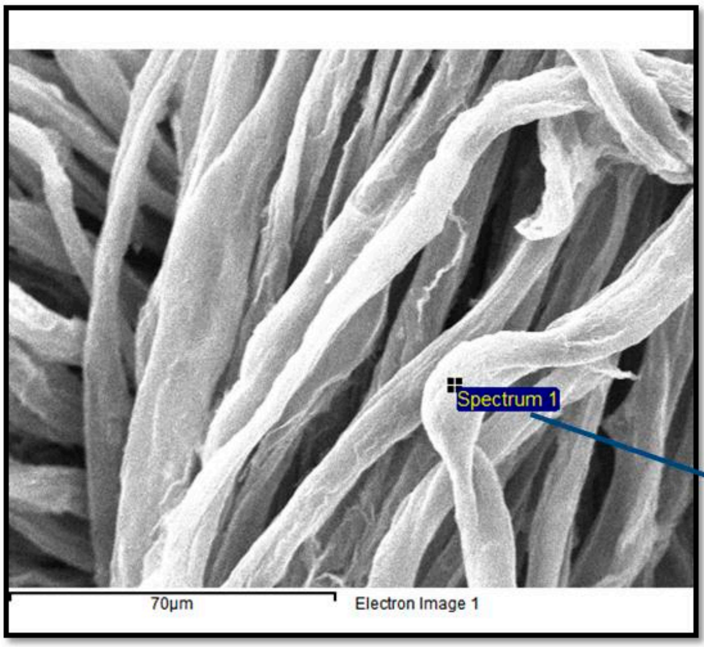

Element	Weight%
C	58.54
O	31.59
Na	1.48
Al	0.99
Si	2.39
P	4.47
Ca	0.54
Totals	100.00

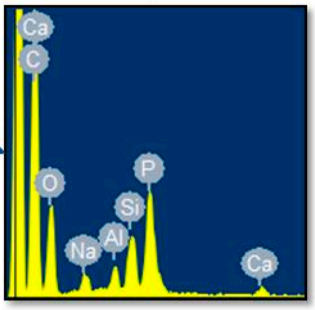

Figure S5. EDS analyses performed on 5% APP/MTM residues collected at the end of cone calorimetry tests.
